# The Physical Reconstruction of American Soldiers

**Published:** 1918-12

**Authors:** 


### THE PHYSICAL RECONSTRUCTION OF AMERICAN SOLDIERS

After hearing the plans for the re-education and physical reconstruction of the disabled soldiers in the United States Army, as described by Colonel Frank Billings at the recent meeting of the Research Committee, no A. E. F. medical officer present could help feeling a sense of pardonable pride in being a citizen of a nation that makes such provision for sons who are wounded in battle or otherwise disabled in the service of their country. Nor could he also fail to feel pride in the medical profession of a country that can produce a man with such broad vision and genius for organization as everyone accords to Colonel Billings, — a reputation that is justified by his past achievements and by his success in the great and altruistic work in which he is now engaged. It is unquestionably largely through the efforts and the diplomacy of Colonel Billings that the work of restoring disabled soldiers to lives of usefulness and happiness is being carried out on a scale and in a way that was never dreamed of during and after previous wars in which the United States has been engaged.

Colonel Billings pointed out that, before soldiers who have disabilities acquired in line of duty have been discharged from service, they will be cured both physically and functionally as early as conditions will permit. The disabled soldier will be kept in the army until he is able to return to his pre-war vocation, or until he is a wage earner, and therefore independent in some other line of work to which he is adapted, in spite of the disability, and for which he will have the best training that the resources of the United States can secure.

It is interesting to note that the instructors in these training centres are chiefly disabled soldiers; 90 o/o in 16 hospitals according to Colonel Billings. The draft law, which represents the soul of a democratic government in war, brought into the army men from every vocation in life. It, therefore, is providing for the training of those who have been disabled serving their country. Many disabled soldiers who are experienced mechanicians or skilled in various lines of work may not be able for several months to go back to the vocation in which they were engaged prior to the war, but they may have the strength to spend several hours a day teaching their trades or professions to others.

The plans for physical reconstruction which have been worked out by Colonel Billings and his confreres do not conflict with the work of any specialty of the medical department of the American Expeditionary Force; but, on the contrary, as Colonel Billings has explained, the co-operation of every medical man from the surgeon at the front to the medical officer at the port is needed and desired to make the work of physical reconstruction a success. Every medical officer who has charge of a disabled soldier should consider it a duty to make the effort to restore function and to teach him that his wounds or illness will not render him helpless and dependent even if he cannot return to his former vocation.

The Chief Surgeon has approved Colonel Billings' plan to send reconstruction aids in physio-therapy and occupational therapy to each base hospital in France to assist the surgeons in the work of re-educating soldiers whose wounds or illnesses are such that they cannot be returned to the line. These specially trained men and women are expected to work under the direction of the surgeon in charge of the case, and they will, no doubt, be helpful in dealing with severely wounded men who are naturally depressed by the fact that they cannot go back into the line to fight, or by the fear that they may be dependents for the rest of their lives.

This work of the physical reconstruction of disabled soldiers is a wonderful advance over the old pension system. It is not only of economic importance to the United States in making men independent as wage earners who otherwise would have to be supported by the government; it is an altruistic movement to add to the comfort and happiness of the gallant men who have been disabled in service. It will also supply to many vocations the needed personnel to take the places of the heroes who have made the supreme sacrifice for their country.

